# A Better Fruit Quality of Grafted Blueberry Than Own-Rooted Blueberry Is Linked to Its Anatomy

**DOI:** 10.3390/plants13050625

**Published:** 2024-02-24

**Authors:** Bo Zhu, Peipei Guo, Shuangshuang Wu, Qingjing Yang, Feng He, Xuan Gao, Ya Zhang, Jiaxin Xiao

**Affiliations:** Anhui Provincial Key Laboratory for the Conservation and Utilization of Important Biological Resources, College of Life Sciences, Anhui Normal University, Wuhu 241000, Chinahf_cf@ahnu.edu.cn (F.H.); gaoxuan@ahnu.edu.cn (X.G.)

**Keywords:** interspecific grafting, ‘O’Neal’ blueberry, fruit quality, anatomic structure, drought resistance

## Abstract

To further clarify the impact of different rootstocks in grafted blueberry, fruit quality, mineral contents, and leaf gas exchange were investigated in ‘O’Neal’ blueberry (*Vaccinium corymbosum*) grafted onto ‘Anna’ (*V. corymbosum*) (AO), ‘Sharpblue’ (*V. corymbosum*) (SO), ‘Baldwin’ (*V. virgatum*) (BO), ‘Plolific’ (*V. virgatum*) (PO), and ‘Tifblue’ (*V. virgatum*) (TO) rootstocks and own-rooted ‘O’Neal’ (NO), and differences in anatomic structures and drought resistance were determined in AO, TO, and NO. The findings revealed that fruit quality in TO and PO was excellent, that of BO and SO was good, and that of AO and NO was medium. ‘Tifblue’ and ‘Plolific’ rootstocks significantly increased the levels of leaf phosphorus and net photosynthetic rate of ‘O’Neal’, accompanied by a synchronous increase in their transpiration rates, stomatal conductance, and intercellular CO_2_. Additionally, the comprehensive evaluation scores from a principal component analysis based on anatomic structure traits from high to low were in the order TO > AO > NO. The P_50_ (xylem water potential at 50% loss of hydraulic conductivity) values of these grafted plants descended in the order NO > AO > TO, and the branch hydraulic conductivity of TO and sapwood hydraulic conductivity of TO and AO were significantly lower than those of NO. Thus, TO plants exhibited the strongest drought resistance, followed by AO, and NO, and this trait was related to the effects of different rootstocks on the fruit quality of ‘O’Neal’ blueberry. These results provided a basis for a deeper understanding of the interaction between rootstocks and scions, as well mechanisms to improve blueberry fruit quality.

## 1. Introduction

Blueberry (*Vaccinium* spp.), from the family Ericaceae, is a perennial fruit tree that bears small berries with a delicate taste and moderate sweetness. The fruit can be processed for use in food products or eaten fresh and is rich in vitamins and anthocyanins [1,2], which are beneficial in combatting certain human cancers, reducing oxidative stress in organs, regulating blood sugar levels, supporting gut microbiota, and decreasing inflammatory responses [3,4,5]. Blueberry ranks first among the 15 global healthy foods listed by British nutritionists and is also one of the five major human dietary supplements listed by the Food and Agriculture Organization of the United Nations [6,7]. Blueberry has a slender root system, with no root hairs, shallow distribution, and strict soil requirements, which usually requires loose soil, low pH, and high organic matter content [8]. Species such as highbush blueberry (*V. corymbosum* L.), lowbush blueberry (*V. chamaebuxus* C.Y.Wu), and rabbiteye blueberry (*V. virgatum* Ait.) are cultivated [9]. Rabbiteye and southern highbush (SHB) blueberries are suited to the conditions (temperature, light, and precipitation) found in Anhui Province, China, an area located in the northern subtropical zone. Rabbiteye exhibits vigorous growth, strong adaptability, high yield, and fruit of moderate quality, which matures during the plum rainy season, making picking, storage, and transportation inconvenient. SHB fruit is of superior quality, with a thick wax layer. Although the vigor of SHB shrubs is relatively weak, they have moderate fruit-bearing capacity and are more drought resistant than rabbiteye shrubs. SHB fruits reach maturity during mid- to late-May, about 1 month earlier than rabbiteye berries [10]. Thus, a grafting SHB species (scion) onto a rabbiteye species (rootstock) may be useful for production.

For commercial production, most fruit trees are grafted and replanted to renew species. The rootstock can affect the water use efficiency, scion nutrition, and fruit quality of grafted trees [11,12]. For example, four citrus scions grafted on trifoliate oranges were shown to produce fruit of higher quality than those grafted on Hongju [13]. Additionally, in poplars, interspecific grafting produces superior quality trees, mainly due to improvements in photosynthesis, the biosynthesis of secondary metabolites, plant hormone regulation, etc. [14]. Considering yield, fruit quality, and mineral nutrient uptake, ‘Carrizo’ and ‘Troyer’ citranges make suitable rootstocks for ‘Rio Red’ grapefruit trees grown in the Mediterranean region [15]. In peach, medium to high vigor plum-based hybrid rootstocks promote better fruit quality, based on a higher concentration of total soluble solids and other yield attributes, demonstrating their commercial value as novel rootstocks for this fruit crop [16]. Therefore, rootstocks can regulate plant growth and development and improve fruit quality by affecting the concentrations of mineral elements in grafted plants. In addition, grafting is a proven and effective tool to enhance plant drought resistance ability by regulating their physiological and molecular processes [17,18]. Reported mechanisms for drought resistance of grafted plants can bring about modifications in root traits, scion-rootstock communication, and scion morpho-physiological characteristics [18,19,20]. 

Grafting and replanting methods are not widely used in blueberry production, as the effects of these methods on crop quality and stress resistance have not been fully evaluated to date, although the fruit yield and quality of SHB ‘Sharpblue’ grafted on *V. bracteatum* Thunb. was demonstrated to be significantly improved compared with own-rooted ‘Sharpblue’ [7], ‘Sparkleberry’-grafted SHB ‘Patrecia’ produces larger berries than own-rooted plants [21], and interspecific grafted blueberry has higher levels of sugars and secondary metabolites as compared to intraspecific grafted blueberry [22]. For this study, we selected ‘O’Neal’ blueberry grafted onto different rootstocks to compare and analyze differences in fruit quality, photosynthesis, mineral nutrition, anatomic structure, and drought resistance. We predicted that, fruit quality traits, gas exchange characteristics and mineral nutrients of grafted and own-rooted blueberry shrubs were linked to their anatomy. In particular, we hypothesized that interspecific grafting (‘Tifblue’-grafted ‘O’Neal’) had better fruit quality than intraspecific grafting (‘Anna’-grafted ‘O’Neal’) and own-rooted ‘O’Neal’, and this was related to the optimization of blueberry photosynthesis efficiency, mineral nutrients, anatomical structure, and improved drought resistance. This study will provide a new perspective for understanding the interaction between scions and rootstocks and offer a scientific basis for the development of blueberry propagation through grafting to improve fruit quality. 

## 2. Results

### 2.1. Fruit Quality

As shown in Table 1, on 23 May (56 DAFB), the berry weight of TO was significantly higher than that of AO, SO, and BO, but comparable to that of PO and NO. The longitudinal and transverse diameters of AO and transverse diameters of BO, showed no significant difference from NO, the fruit yield of AO and SO were significantly smaller than that of NO, but the longitudinal and transverse diameters and fruit yield of TO and PO were significantly greater than those of NO. The total soluble solids (TSS) concentration of NO fruits was the lowest and significantly lower than that of grafted ‘O’Neal’ berries, while that of TO fruit was the highest. The titratable acidity (TA) of NO fruit was relatively high, with no significant difference from that of AO and BO fruit, and was significantly higher than that of TO, SO, and PO. The solid:acid ratio of TO and PO fruit was relatively high, followed by SO and BO fruit, while the solid:acid ratio of NO fruit was the lowest and significantly lower than that of grafted ‘O’Neal’ berries. The Vitamin C content of NO fruit was the highest and significantly higher than that of grafted ‘O’Neal’ berries. The anthocyanin contents were highest to lowest in the order: TO > PO > SO > NO > AO.

Referring to the weight values of economic traits provided by Li [23], weight values were assigned to various quality traits of the blueberries. The weight values 0.4, 0.2, 0.1, and 0.1 were assigned to the TSS, fresh weight, anthocyanins, and solid:acid ratio, respectively. TA, vitamin C concentration, and longitudinal and transverse diameters were all assigned weight values of 0.05. The comprehensive evaluation values are shown in Table 2. The fruit quality of TO and PO was generally excellent (with a comprehensive evaluation value > 0.65), that of BO and SO was generally good (with a comprehensive evaluation value of 0.45~0.65), while that of AO and NO was comprehensively medium (with a comprehensive evaluation value of 0.25~0.45).

Based on the measurements of eight quality traits, the eigenvectors, eigenvalues, variance contribution rates, and cumulative variance contribution rates related to the original data were obtained through principal component analysis. The results are shown in Table S1. The cumulative variance contribution rate of the first two principal components reached 82.870%, so the first two principal components were used for a comprehensive evaluation of blueberry fruit quality. The ranking of principal component scores was in the order: TO, PO, SO, BO, AO, NO (Table 3). 

### 2.2. Photosynthesis 

*P_n_* and *T_r_* for TO and PO were relatively high, significantly higher than the corresponding values for NO and grafted ‘O’Neal’ plants, while the *P_n_* and *T_r_* values of SO, BO, and AO were not significantly different from those of NO. The *G_s_* value for PO was significantly higher than that of NO, while the *G_s_* values for AO, SO, BO, and TO were not significantly different from NO. The *C_i_* in TO and PO were significantly higher than those in NO and other grafted ‘O’Neal’ plants, while the *C_i_* in AO was significantly lower than that in NO (Table 4). *P_n_* showed a highly significant positive correlation with *T_r_*, *G_s_*, and *C_i_*; *T_r_* and *C_i_* showed a highly significant positive correlation and also exhibited a significant positive correlation with *G_s_*; and *G_s_* showed a highly significant positive correlation with *C_i_* (Table S2).

Apart from the lack of a significant difference in the chlorophyll a concentration between SO and NO, the concentrations of chlorophyll a in other grafted plants were significantly higher than that in NO. There were no significant differences in the chlorophyll b and carotenoid concentrations or the a/b ratio between grafted ‘O’Neal’ plants and NO. The chlorophyll a + b concentration of each grafted plant was significantly higher than that of NO (Table S3).

### 2.3. Mineral Nutrients

**Figure 1:**
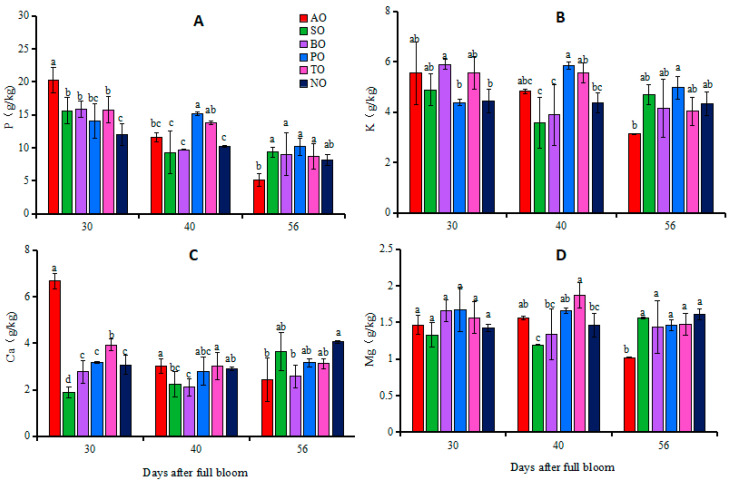
Changes in phosphorus (**A**), potassium (**B**), calcium (**C**) and magnesium (**D**) concentrations in leaves of grafted and own-rooted ‘O’Neal’. Note: AO, SO, BO, PO, and TO represent ‘O’Neal’ grafted onto ‘Anna’, ‘Sharpblue’, ‘Baldwin’, ‘Plolific’, and ‘Tifblue’, respectively; NO represents own-rooted ‘O’Neal’. Error bars were calculated based on three replicates; Different lowercase letters indicate significant differences with *p* < 0.05, as determined by Tukey’s test.

At 30 DAFB, there was no significant difference in leaf P concentrations between PO and NO, while the leaf P concentrations of other grafted ‘O’Neal’ leaves were significantly higher than that of NO. There was no significant difference in K and Mg concentrations between the leaves of grafted ‘O’Neal’ and own-rooted plants. The Ca concentrations in AO and TO leaves were significantly higher than that in NO, while in SO leaves the Ca concentration was significantly lower than that in NO. There was no significant difference in leaf Ca concentrations among BO, PO, and NO leaves. At 40 DAFB, P concentrations in PO and TO leaves and K concentration in PO leaves were significantly higher than those in NO, while there was no significant difference in P and K concentrations between the other grafted plants and NO. The Ca concentration in BO leaves was significantly lower than that in NO, while the Mg concentration in TO leaves was significantly higher than that in NO, but there was no significant difference in Ca and Mg concentrations between the leaves of other grafted ‘O’Neal’ and own-rooted plants. At 56 DAFB, there were no significant differences in the P and K concentrations in the leaves of each grafted plant and NO. Except for the significantly lower concentrations of Ca and Mg in AO and BO leaves compared to NO, there was no significant difference in Ca and Mg in the leaves of the other grafted plants and NO (Figure 1). 

**Figure 2:**
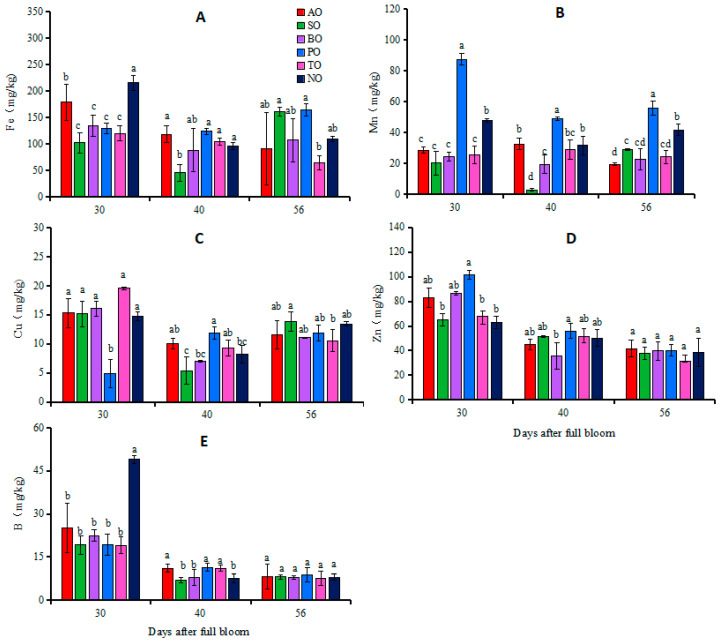
Changes in iron (**A**), manganese (**B**), copper (**C**), zinc (**D**) and boron (**E**) concentrations in leaves of grafted and own-rooted ‘O’Neal’. Note: AO, SO, BO, PO, and TO represent ‘O’Neal’ grafted onto ‘Anna’, ‘Sharpblue’, ‘Baldwin’, ‘Plolific’, and ‘Tifblue’, respectively; NO represents own-rooted ‘O’Neal’. Error bars were calculated based on three replicates; Different lowercase letters indicate significant differences with *p* < 0.05, as determined by Tukey’s test.

At 30 DAFB, the Fe concentration in NO leaves was the highest, followed by AO, whereas the Fe concentrations in the other grafted ‘O’Neal’ plants were relatively low. The Mn concentration in PO leaves was significantly higher than that in NO, while in the other grafted plants, the Mn contents were significantly lower than that in NO. Except for the significantly lower Cu concentration in PO leaves compared to NO, there was no significant difference in the Cu concentrations between the other grafted plants and NO. The Zn concentration in PO leaves was significantly higher than that in NO, while there was no significant difference in Zn concentrations between the other grafted plants and NO. The leaf B concentration in each grafted plant was significantly lower than that in NO (Figure 2). 

At 40 DAFB, apart from the significantly lower Fe concentration in SO leaves, there was no significant difference in the Fe concentrations between the other grafted plants and NO. PO leaves exhibited the highest Mn concentration, followed by NO, TO, and AO leaves, and finally SO leaves, which had the lowest concentration of Mn. The Cu concentration in PO leaves was significantly higher than that in NO, while there was no significant difference in leaf Cu concentrations between the other grafted plants and NO. There was no significant difference in leaf Zn concentrations between grafted plants and own-rooted plants. The B concentrations in AO, PO, and TO leaves were significantly higher than that in NO, while there was no significant difference in the B concentrations of SO or BO leaves compared to NO (Figure 2). 

At 56 DAFB, there was no significant difference in the leaf Fe concentrations of each grafted plant and NO. Except for the significantly higher Mn concentration in PO leaves compared to NO, in other grafted plants, this was significantly lower than that in NO. There was no significant difference in concentrations of Cu, Zn, or B in the leaves of any grafted plants compared to own-rooted plants (Figure 2).

### 2.4. Anatomic Structure of Leaves and Stems

**Figure 3:**
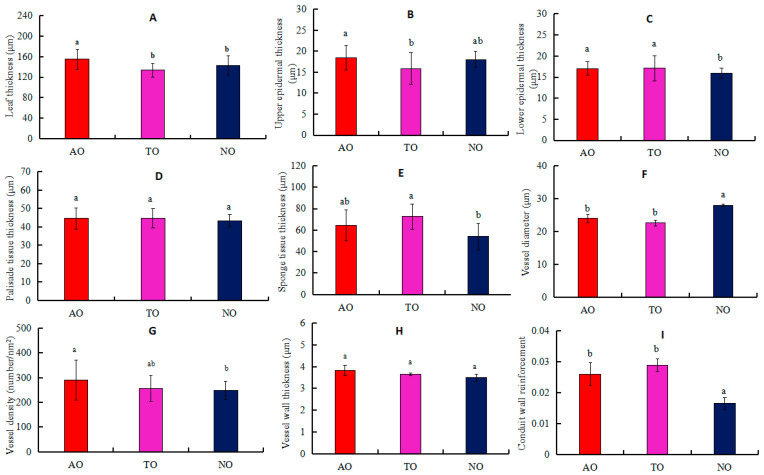
Effects of different rootstocks on the anatomical structure in leaves and stems of grafted and own-rooted ‘O’Neal’. Note: AO and TO represent ‘O’Neal’ grafted onto ‘Anna’ and ‘Tifblue’ rootstocks, respectively; NO represents own-rooted ‘O’Neal’. In panels (**A**–**E**), the leaf thickness (LT), upper epidermal thickness (UET), lower epidermal thickness (LET), palisade tissue thickness (PTT), and sponge tissue thickness (STT) are represented; In panels (**F**–**I**), stem xylem vessel diameter (Dv), vessel density (VD), vessel wall thickness (VT), and conduct wall reinforcement (CWR) are shown. Different lowercase letters indicate significant differences with *p* < 0.05, as determined by Tukey’s test.

The LT values for AO plants were significantly higher than those for TO and NO, while there was no significant difference in LT between TO and NO (Figure 3A). The UET of AO plants was significantly higher than that of TO, while there was no significant difference in UET between AO and TO (Figure 3B). LET values for AO and TO were significantly higher than that of NO, while there was no significant difference in LET between AO and TO (Figure 3C). STT in TO was significantly higher than that in NO, while there was no significant difference in STT between TO and AO (Figure 3E). The Dv measurement in NO was significantly higher than that in AO and TO, but there was no significant difference in the Dv values between AO and TO (Figure 3F). The VD of TO was not significantly different from that of AO and NO, but the VD of AO was significantly higher than that of NO (Figure 3G). There was no significant difference in the CWR value between TO and AO, but this measurement was significantly higher in TO and AO than NO (Figure 3I). There was no significant difference in PTT or VT among AO, TO, and NO (Figure 3D,H).

### 2.5. Principal Component Analysis of Anatomic Structure Traits

Based on the measurement results of 11 anatomic traits, including LT, UET, LET, PTT, STT, PTT/STT, Dv, VD, VT, CWR, and K_H_, the eigenvectors, eigenvalues, variance contribution rates, and cumulative variance contribution rates related to the original data were obtained through principal component analysis, as shown in Table S4. Based on data for anatomic structure traits in stems and leaves of grafted and own-rooted ‘O’Neal’, using principal component analysis, the average membership function values of each index were obtained, as shown in Table 5. The comprehensive evaluation scores for the stem and leaf anatomical structure traits descended in the order: TO, AO, and NO (Table 5).

### 2.6. Hydraulic Functional Traits of Stems

**Figure 4:**
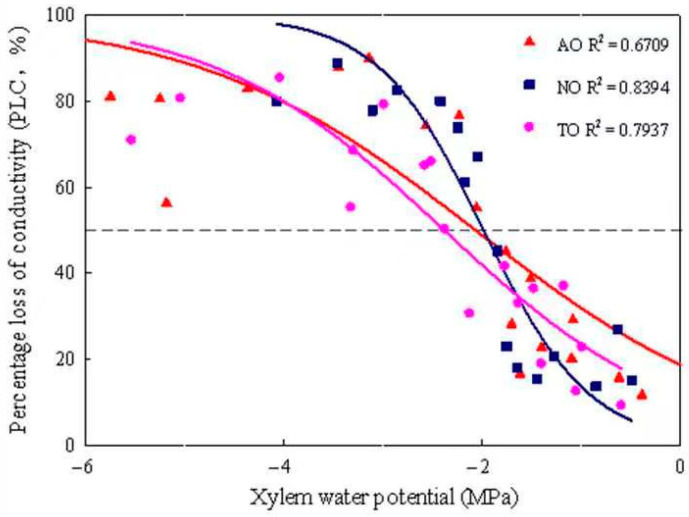
Xylem embolism vulnerability curves for stems from grafted and own-rooted ‘O’Neal’. Note: The curves were established with the bench dehydration method. Different symbols and regression lines with the same color represent three blueberry cultivars. The horizontal dashed line gives 50% loss of hydraulic conductivity occurring in the xylem. AO and TO represent ‘O’Neal’ grafted onto ‘Anna’ and ‘Tifblue’ rootstocks, respectively; NO represents own-rooted ‘O’Neal’.

**Figure 5:**
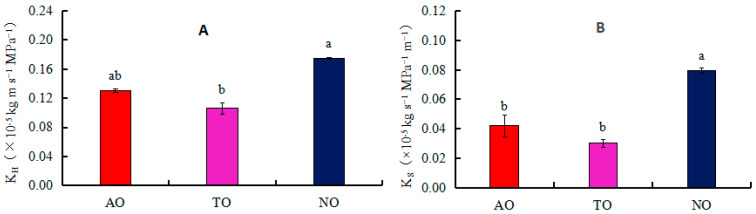
Effects of different rootstocks on branch hydraulic conductivity (K_H_) and sapwood hydraulic conduct (K_s_) of grafted and own-rooted ‘O’Neal’. Note: AO and TO represent ‘O’Neal’ grafted onto ‘Anna’ and ‘Tifblue’ rootstocks, respectively; NO represents own-rooted ‘O’Neal’. In panels (**A**,**B**), the K_H_ and K_s_ are represented. Different lowercase letters indicate significant differences with *p* < 0.05, as determined by Tukey’s test.

The xylem embolism vulnerability curves for stems of AO, TO, and NO exhibited “S” shapes. The P_50_ values of TO, AO, and NO were −2.40 MPa, −2.08 MPa, and −2.00 MPa, respectively (Figure 4); the corresponding P_12_ values were −0.35 MPa, 0.67 MPa, and −0.92 MPa, respectively; and the corresponding P_88_ values were −4.71 MPa, −4.88 MPa, and −3.07 MPa, respectively. The K_H_ of NO was significantly higher than that of TO, while there was no significant difference in K_H_ between AO and TO. The K_S_ of NO was significantly higher than that of AO and TO, but there was no significant difference in Ks between AO and TO (Figure 5).

## 3. Materials and Methods

### 3.1. Plant Materials and Soil Properties

Sampling was conducted at the blueberry orchard (30°47′ N, 118°08′ E) in Gelin Village, Nanling County, Wuhu City, Anhui Province, from April to May in 2021 and 2022. The experimental materials included southern highbush blueberry (SHB) ‘O’Neal’ (*V. corymbosum*) grafted onto ‘Anna’ (*V. corymbosum*) (AO), ‘Sharpblue’ (*V. corymbosum*) (SO), ‘Baldwin’ *V. virgatum*) (BO), ‘Plolific’ (*V. virgatum*) (PO) and ‘Tifblue’ (*V. virgatum*) (TO) rootstocks, as well as own-rooted ‘O’Neal’ (NO). ‘Anna’, ‘Sharpblue’, ‘Baldwin’, ‘Plolific’, ‘Tifblue’, and ‘O’Neal’ bushes were planted in the autumn of 2008, and the same cleft grafting was carried out in spring 2019. When grafting, the grafting site (height from the ground), the diameters of the scion and rootstock are relatively consistent. All grafted shrubs grow well (Figure S1). Nine shrubs from each rootstock/scion combination were used, planted at 2.5 × 1.5 m spacing on a red-yellow loam soil with the following properties: pH 4.4, organic matter 14.1 g kg^−1^, available phosphorus (P) 404 mg kg^−1^, available potassium (K) 339 mg kg^−1^, available calcium (Ca) 268 mg kg^−1^, available magnesium (Mg) 357 mg kg^−1^, available iron (Fe) 293 mg kg^−1^, available zinc (Zn) 30.5 mg kg^−1^, available copper (Cu) 7.21 mg kg^−1^, and available manganese (Mn) 24.4 mg kg^−1^. The experiment was conducted as a randomized block with ten fruits and five leaves on each of the nine bushes of each rootstock/scion combination. All shrubs received the same orchard management program.

### 3.2. Collection of Fruit, Leaf and Stem Samples

The flowers of grafted and own-rooted ‘O’Neal’ reached full bloom on 27 March 2022, and fruits and leaves were collected on April 27, [30 days after full bloom (DAFB)], May 7 (40 DAFB), and May 23 (56 DAFB), respectively. Thirty fruits and fifteen leaves of approximately the same size and free from pests and diseases were randomly picked from three bushes, and bulked as a biological replication and three replicates for each rootstock/scion combination were used for the experiment. The fruits and leaves were transported to the laboratory in an ice box, and stored at 4 °C for future use. 

In addition, the current year’s shoots, and the third mature leaf below the tops of TO, AO, and NO were collected in June/July 2022 for anatomical measurement (2 branches and 3–4 leaves from each tree). Stem segments (approximately 2–3 cm long) were cut from between the 5th and 6th leaves below the top of the cane. The stem segments and leaves were fixed in FAA fixation solution (mixed with 70% ethanol, glacial acetic acid, and formaldehyde in a volume ratio of 18:1:1) for subsequent dissection. Moreover, several of the current year’s shoots from TO, AO, and NO were selected to determine xylem water conductivity and create an embolism vulnerability curve (see below for details).

### 3.3. Fruit Quality Traits

The 90 fresh fruits from each rootstock/scion combination during the harvest season (on 56 DAFB) were weighed, the longitudinal and transverse diameters were measured using 0.02 cm vernier calipers, the total soluble solids content was measured using a hand refractometer, titratable acidity was determined by the potentiometric titration method [24], the solid:acid ratio (the ratio of total soluble solids to titratable acidity) was calculated, the vitamin C concentration was measured using UV spectrophotometry [25], and the anthocyanin concentration was determined using the pH differential method [26]. The fruit yield (g/plant) was obtained by weighing the harvested fruit.

### 3.4. Fuzzy Comprehensive Evaluation of Fruit Quality Traits

According to the method reported by Li [23], extreme value standardization was carried out on various quality traits of the blueberry fruits. The formula for calculating the membership function value for the fuzzy comprehensive evaluation method was as follows: the comprehensive membership function value Xi (u) = [∑Xij (u)]/n, the positive indicator membership function value Xij(u) = (Xij − Xjmin)/(Xjmax − Xjmin), the membership function value of the negative indicator Xij (u) = 1 − (Xij − Xjmin)/(Xjmax − Xjmin), where ∑Xij (u) represents the cumulative membership function value of the j-th indicator of the i-th grafted or own-rooted ‘O’Neal’, Xij represents the measured value of the j-th indicator of the i-th grafted or own-rooted ‘O’Neal’, Xjmin represents the minimum value of the j-th indicator, Xjmax represents the maximum value of the j-th indicator, and n represents the number of indicators. According to the impact of each trait on comprehensive quality, weight values were assigned to each quality trait to obtain a weight matrix (A), with the comprehensive evaluation value (B) calculated as B = A × R, wherer R = Xij (u).

### 3.5. Photosynthetic Parameters

On 14 May 2022, from 8:30 a.m. to 11:30 a.m., photosynthetic parameters such as net photosynthetic rate (*P_n_*), transpiration rate (*T_r_*), stomatal conductance (*G_s_*), and intercellular CO_2_ concentration (*C_i_*) in leaves of grafted and own-rooted ‘O’Neal’ plants were measured using the Li-6400XT portable photosynthesis measurement system (LI-COR Biosciences, Lincoln, NE, USA). To measure photosynthetic parameters, nine fully expanded mature leaves (fourth leaves from the shoot apex) at a height of 1.5 m were selected from each rootstock/scion combination, a temperature of 25 °C, and an open gas path with CO_2_ at a concentration of 400 ± 100 μmol mol^−1^ were used. The built-in light source provided a light intensity of 1200 μmol m^−2^ s^−1^. The photosynthetic pigments of the leaves were extracted using 95% ethanol, and the chlorophyll a, b, a + b and carotenoid concentrations, as well as chlorophyll a/b, were calculated [27].

### 3.6. Mineral Elements

Fruits and leaves were washed with deionized water to remove surface dust, the surface moisture was removed, and the samples were transferred to an oven for 15 min at 105 °C. Then, the oven temperature was adjusted to 70 °C, and the samples were dried to constant weight. The samples were then ground into powder using a mortar. The dried and ground fruits and leaves were digested using the nitric acid perchloric acid (4:1) digestion method, and the digestion solution was determined for P, K, Ca, Mg, Fe, Mn, Cu, Zn, and boron (B) concentrations using an Optimal2100DV inductively coupled plasma emission spectrometer (Pekin Elmer, Waltham, MA, USA).

### 3.7. Morphological Anatomy of Leaves and Stem Segments

The leaves and stem segments kept in FAA fixative were rinsed with clean water and then sectioned using a microtome (Leica RM2016, Shanghai, China) to obtain 10 μm thick cross sections. The sections were stained with the methyl green and safranine (1:1) mixed solution for 5 min, and dehydrated in gradient ethanol solutions (70%, 85%, and 95%) for 30 s before being transferred to slides. The anatomical structures of the leaves and stems were observed and photographed under a light microscope (DMi8, Leica, Wetzlar, Germany). ImageJ software (version 1.50i, National Institutes of Health, Bethesda, MD, USA) was used to process the photos and measure leaf thickness (LT, μm), upper epidermal thickness (UET, μm), lower epidermal thickness (LET, μm), palisade tissue thickness (PTT, μm), sponge tissue thickness (STT, μm), stem xylem vessel diameter (Dv, μm), vessel density (VD, number/nm^2^), vessel wall thickness (VT, μm), and conduit wall reinforcement (CWR). Dv was averaged from two measurements on different axes. VD was calculated as the number of vessels in a selected area of the xylem. VT was determined as the double wall thickness of two neighboring vessels. CWR was calculated as the square of the ratio of vessel wall thickness to Dv. All minimum measurement requirements were based on Scholz et al. [28].

### 3.8. Stem Hydraulic Conductivity

According to Sperry et al. [29] and Zhang et al. [10], the branch hydraulic conductivity (K_H_, kg m s^−1^ MPa^−1^) and sapwood hydraulic conductivity (K_S_, kg s^−1^ MPa^−1^ m^−1^) were measured for AO, TO, and NO on 17 April 2023. Briefly, at least nine fresh branches of each grafted or own-rooted ‘O’Neal’ plants were collected in the early morning, kept in wet black bags, and taken back to the laboratory within 2 h. The branches were then rehydrated under water for 2 h. A segment longer than the maximum vessel length determined by the air injection method [30] was then cut under water and connected to the modified Sperry apparatus to measure the hydraulic conductivity. In brief, a 0.006 MPa pressure from a 10 mM KCl solution was applied to the segment, and the flow rate of the segment (F, μg s^−1^) was determined with a pipette. The hydraulic conductivity of the segment (K_H_) was calculated as K_H_ = F/(P/L), where P represents 0.006 MPa and L represents the length of the segment. Then, the sapwood area of the segment was determined using safranine solution [10]. The sapwood hydraulic conductivity (K_S_) was calculated as the ratio of K_H_ to the sapwood area.

### 3.9. Vulnerability Curves

The bench dehydration method [10,29] was applied to determine the embolism vulnerability curves for AO, TO, and NO. The branch collection and rehydration processes were the same as those mentioned in the ‘stem hydraulic conductivity’ section. The branches were then placed on an experimental table to dry naturally for different periods (0–24 h) to obtain the tension gradient in the xylem. Then, the branches were wrapped completely in a black plastic bag for 0.5~1 h to establish water equilibrium between the leaves and stems. Three leaves from different positions on each branch were cut to measure the water potentials with a pressure chamber (PMS 1505EXP, company, Corvallis, OR, USA), and the average value was taken as the xylem water potential of the branch (Ψ, MPa). A circa 10 cm segment from each branch was cut under water and connect to the modified Sperry apparatus to determine the initial hydraulic conductivity (K_i_, kg m s^−1^ MPa^−1^). Then, the branch segment was flushed with 10 mM KCl solution via the pressure chamber under 0.15 MPa to remove the xylem embolism. The maximum hydraulic conductivity (K_max_, kg m s^−1^ MPa^−1^) was measured, and the percentage loss of hydraulic conductivity (PLC, %) of each segment was calculated as: PLC (%) = 100 × (K_max_ − Ki)/K_max_. The xylem vulnerability curve was then established by plotting the relationship between the xylem water potential and PLC values of different branches, PLC (%) = 100/(1 + exp((S/25)/(Ψ − b))), where S represents the slope of the fitted curve, and b is the xylem water potential when the xylem hydraulic conductivity loss is 50% (P_50_, MPa). Then, the xylem water potential at 12% and 88% loss of xylem hydraulic conductivity, i.e., P_12_ (MPa) and P_88_ (MPa), were calculated as P_12_ = 2/(S/25) + b, and P_88_ = −2/(S/25) + b, respectively [31].

### 3.10. Data Analysis

SPSS 17.0 (IBM, Armonk, New York, NY, USA) was used for principal component analysis, one-way ANOVA and Tukey methods were used to determine significant differences (*p* < 0.05), and the Pearson’s correlation coefficient was used for correlation analysis. SigmaPlot 14.0 (Systat Software Inc., Erkrath, Germany) was used to create graphs.

## 4. Discussion

### 4.1. Rootstocks Affect Scion Fruit Quality

The interaction between rootstocks and scions has been shown to affect the fruit quality of various crops such as tomato [32], cucumber [33], and persimmon [34]. In this study, there were differences in the fruit quality of ‘O’Neal’ blueberry grafted on different rootstocks. The results indicated that, compared with own-rooted ‘O’Neal’, all five rootstocks significantly increased TSS concentrations, solid:acid ratios, and anthocyanin concentrations (except for ‘Anna’ rootstock) in fruit. The results of the fuzzy comprehensive evaluation and the principal component analysis showed that ‘Tifblue’-grafted ‘O’Neal’ had the best comprehensive fruit quality, while ‘Anna’-grafted and own-rooted ‘O’Neal’ had relatively poor comprehensive fruit quality. This result was basically consistent with the results of the research conducted in 2021 (Tables S5 and S6). on the other hand, interspecific grafting (‘Tifblue’ and ‘Plolific’-grafted ‘O’Neal’) had higher fruit yield and plant height than own-rooted ‘O’Neal’, while intraspecific grafting (‘Anna’ and ‘Sharpblue’-grafted ‘O’Neal’) had lower fruit yield and plant height than own-rooted ‘O’Neal’ (Table 1 and Table S7), and this had a certain correspondance with the fruit quality. Similarly, the growth vigor, early bearing and productivity and fruit quality of Wufanshu-grafted ‘Sharpblue’ blueberry were significantly improved [7]. Citrus scions on *Poncirus trifoliata* rootstock had better fruit quality with higher TSS and lower TA compared to those of on Hongju rootstock, and Cre-miR399-3 p may be one of the important factors regulating the fruit quality in *Poncirus trifoliata* rootstock [13]. 

Fruit quality, e.g., sugar accumulation, is closely related to leaf photosynthesis. Factors affecting plant photosynthesis rates include stomatal and non-stomatal limiting factors [35,36]. The results of this study indicated that both ‘Tifblue’ and ‘Plolific’ rootstocks significantly increased the *P_n_* of grafted plants, accompanied by a synchronous increase in *T_r_*, *G_s_*, and *C_i_*. Correlation analysis also showed that *P_n_* was significantly positively correlated with *T_r_*, *G_s_*, and *C_i_*, while *T_r_* was significantly positively correlated with *C_i_* and *G_s_*. These results further indicated that the increase in the *P_n_* of ‘O’Neal’ grafted on ‘Tifblue’ and ‘Plolific’ rootstocks was mainly influenced by stomatal limitations. In addition, the concentrations of chlorophyll a+b and chlorophyll a (except for ‘Sharpblue’ rootstock) in grafted ‘O’Neal’ were significantly higher than those in own-rooted ‘O’Neal’ (Table S3). Therefore, it was speculated that the increase in the *P_n_* of ‘O’Neal’ triggered by ‘Tifblue’ or ‘Plolific’ rootstock may be influenced not only by stomatal limiting factors but also by non-stomatal limiting factors such as chlorophyll. This was also consistent with research results obtained from 2021 (Tables S8–S10). A similar response was found for watermelon grafted onto bottle gourd rootstock, which promoted photosynthesis by the activation of stomatal and non-stomatal abilities, especially the regulation of a variety of photosynthetic enzymes including Rubisco in grafted plants under NaCl stress [37]. Grafting muskmelon on interspecific rootstocks enhanced photosynthesis and translocation of sugars in muskmelon leaves [38]. 

Individuals with different combinations of rootstocks and scions not only differ in growth and development but also in their abilities to absorb and transport mineral elements [39], as reported in horticultural crops such as citrus [40], apple [41], and cucumber [42]. Fazio et al. [43] pointed-out that rootstocks could induce the absorption of one element, thereby affecting the absorption of other elements. Due to the interaction between rootstocks and scions, there may be differences in the structure and function of the stem xylem, affecting the absorption, transportation, and accumulation of mineral elements. The absorption and accumulation of N, Ca, and Mg in leaves was previously shown to differ between two cucumber cultivars, ‘Jinlu 21-1′ and ‘Jinyan 4′, grafted on ‘black seed’ pumpkin rootstock [44]. In apple, ‘G.890′, the most vigorous rootstock, resulted in lower N and higher K contents in the leaves of ‘Honeycrisp’ apple, while ‘B.9’, the least vigorous rootstock, resulted in lower K and higher N contents [12]. The results of this study showed that during fruit development, compared with own-rooted ‘O’Neal’, ‘Tifblue’ rootstock significantly increased P, Mg, and B concentrations in leaves of ‘O’Neal’, ‘Plolific’ rootstock increased leaf P, K, Mn, and B concentrations, and ‘Anna’ rootstock increased leaf B concentrations, while ‘Sharpblue’ rootstock decreased leaf Fe and Mn concentrations, and ‘Baldwin’ rootstock decreased leaf Ca concentrations (Figure 1 and Figure 2). These results indicated that different rootstocks had varying effects on the mineral nutrient levels in ‘O’Neal’ leaves. Of course, there is also a mutual promotion or inhibition relationship between mineral elements in leaves and fruits, and this correlation can change depending on the rootstock used, as shown in Tables S11–S14. The results showed that the correlation between mineral elements and the correlation between fruit and leaf traits of ‘O’Neal’ grafted on different rootstocks differed. 

In terms of the appropriate range of nutrients for SHB applicable to the United States (P 0.9–1.1 g kg^−1^, K 4.4–7.2 g kg^−1^, Ca 6.2–7.3 g kg^−1^, Mg 1.5–2.7 g kg^−1^, Fe 90–100 mg kg^−1^, Mn 186–253 mg kg^−1^, Zn 22–116 mg kg^−1^, Cu 6–11 mg kg^−1^, and B 14–27 mg kg^−1^) [45], it can be seen that at 40 DAFB, the Ca, Mn, and B concentrations in the leaves of grafted and own-rooted ‘O’Neal’ were low, as were the levels of leaf K and Mg in SO and BO, along with Fe in SO leaves, while the concentrations of nutrients in the leaves of the other grafted plants were at moderate or high levels (Figure 1 and Figure 2). The research results from 2021 showed that the leaves of ‘O’Neal’ grafted onto ‘Anna’, ‘Tifblue’, and ‘Plolific’ rootstocks all contained low levels of Fe, Mn, Cu, Zn, and B (Figures S2 and S3). Therefore, for commercial production, attention should be paid to adding trace element fertilizers such as Mn and B to grafted ‘O’Neal’ plants. 

From the perspective of soil physicochemical properties, the red soil, with a pH of 4.4, was suited to blueberry growth, but the soil organic matter content (14.1 g kg^−1^) was significantly low in the area of study [46]. Therefore, while increasing the application of organic fertilizer to improve soil organic matter content, targeted fertilization should also be carried out based on the requirements of different species or individual grafted plants. For example, increased application of Mn, B, Fe, K, and Mg fertilizers during fruit development may be useful. Similarly, attention should be paid to increasing organic fertilizer with the complement of Fe, Zn, and Mg fertilizer during soil improvement [46,47]. 

### 4.2. Rootstocks Affect Scion Anatomical Structure and Drought Resistance

The above mentioned results indicated that the interaction between rootstock and scion may lead to differences in the anatomic structure and function of stems and leaves. There have been many reports on the effects of rootstocks on the morphological and physiological characteristics of scions in fruit trees such as apple (*Malus pumila* Mill) [48] and grape (*Vitis vinifera* L.) [49]. The anatomical characteristics of leaves are closely related to the adaptability of plants to external environments. Common morphological features related to drought resistance include LT, LET, and STT [50,51]. The results of this study showed that the LT and UET of AO were significantly higher than those of TO. Both ‘Anna’ and ‘Tifblue’ rootstocks significantly increased the LET of ‘O’Neal’. Furthermore, the *P_n_* of TO was significantly higher than those of AO and NO. These findings indicate that a thicker upper epidermis may slow the rate of CO_2_ transportation into leaves, which may hinder the photosynthetic process and thus reduce the *P_n_*. Previously, Jiao et al. [52] found that low temperatures increased the LT of tomatoes, thereby reducing the *P_n_*. There was no significant difference in the STT of leaves between TO and AO, but STT was significantly higher in TO and AO than in NO. This was consistent with the finding that the *P_n_* of grafted ‘O’Neal’ was higher than that of own-rooted ‘O’Neal’. Greater STT is beneficial to increasing the space for gas exchange during photosynthesis, thereby improving photosynthetic efficiency [19].

Vessel elements are the basic structural units of plant xylem that transport water and mineral nutrients, and their diameters are related to water transport efficiency [53]. Dv is also related to xylem embolism vulnerability. Embolism vulnerability can be represented by vulnerability curves, which show how the percentage of embolism in xylem tissue changes according to xylem pressure [54]. Most scholars use the P_50_ value (the xylem water potential value at 50% loss of K_H_) to represent embolism vulnerability [55,56].

Dv largely determines the K_H_ and K_S_. According to the Hagen-Poisson equation, water conductivity is positively correlated with the fourth power of the Dv. The smaller the Dv, the lower its water conductivity, but the stronger its ability to resist air embolism, i.e., there is a trade-off between the “efficiency” (water transport efficiency) and “safety” (embolism resistance) of the xylem [57]. Levionnois et al. [58] indicated that among 26 tropical rainforest tree species, the species with larger Dv values were more vulnerable to embolism. The results of the present study showed that the Dvs of grafted and own-rooted ‘O’Neal’ plants descended in the following order: NO > AO > TO. 

VT can enhance the negative pressure resistance of vessel elements and the mechanical support of xylem conducting tissues, while also enhancing water cohesion inside the vessel and improving drought resistance [59]. CWR is the square of the ratio of vessel wall thickness to Dv. The larger the CWR, the higher the vessel safety performance, and the stronger the resistance of xylem to embolism [60]. Although there was no significant difference in the CWR, K_H_, and K_S_ values of the stems of ‘O’Neal’ grafted onto ‘Tifblue’ and ‘Anna’ rootstocks, the CWR of TO and AO was significantly higher than that of NO, while the K_S_ and K_H_ values of TO were significantly lower than those of NO. This further indicated that the drought resistance of ‘O’Neal’ grafted onto ‘Tifblue’ and ‘Anna’ rootstocks was relatively higher than that of NO.

VD is an important factor affecting the safety of water transportation in vascular plants. It can increase the mechanical support capacity of branches and can avoid loss of function of the entire transport system due to a partial duct blockage. In a study of the relationship between root anatomical structure and drought resistance in *Lespedeza davurica* [61] and *Medicago sativa* [62], varieties with higher VD displayed stronger drought resistance. The results of the present study revealed that, although there was no significant difference in the VD values of AO and TO, they were significantly higher than that of NO, further indicating that the drought resistance of grafted ‘O’Neal’ was greater than that of own-rooted ‘O’Neal’. Similarly, Li [63] explored differences in the xylem structures of six drought-resistant tree species, demonstrating that VD is negatively correlated with embolism vulnerability. Higher VD and lower P_50_ values reduce the likelihood of plant embolism and increase drought resistance. In this study, the P_50_ values of the grafted plants descended in the order: NO, AO, and TO. Thus, the anti-embolism abilities of these plants were in the order: TO > AO > NO. 

Xia et al. [64] pointed-out in their research on the embolism vulnerability of *Camellia* and *Rhododendron hybridum* that *Camellia*, with stronger drought resistance, had significantly greater vessel wall thickness, xylem thickness, and phloem thickness than *Rhododendron hybridum*. Referring to the relevant literature [65,66], combined with the results of this study, indicators such as LT, UET, LET, STT, and PTT, along with Dv, VD, VT, and CWR, are highly correlated with the drought resistance of blueberry. Using the membership function method of principal component analysis, the related indices of grafted and own-rooted ‘O’Neal’ were comprehensively evaluated. The results (Table 5) showed that ‘O’Neal’ grafted onto ‘Tifblue’ rootstock (interspecific grafting) had the strongest drought resistance, followed by ‘O’Neal’ grafted onto ‘Anna’ rootstock (intraspecific grafting), while own-rooted ‘O’Neal’ had the weakest drought resistance.

In summary, excellent blueberry rootstock varieties could improve fruit quality. Interspecific grafting with relatively distant genetic relationships (such as ‘O’Neal’ grafted on ‘Tifblue’ rootstock) had better fruit quality than intraspecific grafting with closer genetic relationships (such as ‘O’Neal’ grafted on ‘Anna’ rootstock), and this was related to the optimization of blueberry photosynthetic efficiency, mineral nutrients, anatomic structure, and improved drought resistance. However, further in-depth research is required to reveal the internal mechanisms associated with these improvements in blueberry traits.

## Figures and Tables

**Table 1:** Effects of different rootstocks on the fruit quality of ‘O’Neal’ blueberry.

Rootstock	Fresh Weight (g/Fruit)	Longitudinal Diameter (mm)	Transverse Diameter (mm)	Fruit Yield (g/Plant)	Total Soluble Solids (%)	Titratable Acidity (%)	Solid:Acid Ratio	Vitamin C (mg/100 g)	Anthocyanin (mg/100 g)
Anna	1.39 ± 0.33 b	10.30 ± 0.37 bc	12.71 ± 0.96 ab	446.67 ± 31.63 d	10.37 ± 0.45 d	0.72 ± 0.12 ab	16.53 ± 0.23 c	673.61 ± 1.36 b	23.35 ± 0.34 f
Sharpblue	1.47 ± 0.05 b	11.27 ± 1.02 ab	13.11 ± 0.75 a	610.33 ± 72.13 c	11.73 ± 0.49 cd	0.40 ± 0.19 bc	23.71 ± 1.78 b	505.99 ± 0.49 d	49.88 ± 0.24 d
Baldwin	1.57 ± 0.09 b	10.64 ± 0.83 ab	12.52 ± 1.11 ab	899.67 ± 31.90 ab	12.97 ± 0.38 bc	0.61 ± 0.11 ab	22.13 ± 0.63 b	510.83 ± 0.38 c	51.61 ± 0.09 c
Plolific	1.60 ± 0.09 ab	11.60 ± 0.47 a	13.77 ± 0.47 a	956.67 ± 27.10 a	13.63 ± 0.78 b	0.39 ± 0.24 bc	33.83 ± 0.69 a	356.62 ± 0.51 e	81.50 ± 1.02 b
Tifblue	1.83 ± 0.02 a	11.66 ± 0.21 a	13.41 ± 1.68 a	966.333 ± 41.68 a	15.70 ± 0.53 a	0.20 ± 0.09 c	35.09 ± 1.03 a	313.61 ± 0.57 f	102.68 ± 0.89 a
Own-rooted	1.39 ± 0.03 ab	9.39 ± 0.41 c	11.75 ± 1.02 b	829.67 ± 101.36 b	8.93 ± 0.57 e	0.85 ± 0.17 a	12.97 ± 0.93 d	675.69 ± 1.51 a	47.06 ± 0.74 e

Note: The data are means of three biological replicates ± standard deviation; Values followed by different lowercase letters within a column are significantly different at *p* < 0.05.

**Table 2:** Comprehensive evaluation on fruit quality traits of grafted and own-rooted ‘O’Neal’.

Grafted and Own-Rooted ‘O’Neal’	Comprehensive Evaluation Value	Quality Level
AO	0.348	Medium
SO	0.491	Good
BO	0.501	Good
PO	0.686	Excellent
TO	0.891	Excellent
NO	0.295	Medium

Note: AO, SO, BO, PO, and TO represent ‘O’Neal’ grafted onto ‘Anna’, ‘Sharpblue’, ‘Baldwin’, ‘Plolific’, and ‘Tifblue’, respectively; NO represents own-rooted ‘O’Neal’.

**Table 3:** Principal component scores and comprehensive evaluation of fruit from grafted and own-rooted ‘O’Neal’.

Grafted and Own-Rooted ‘O’Neal’	Principal Component Score		Synthesis Score	Ranking
	P1	P2		
AO	−5.56	−0.65	−3.98	5
SO	0.20	−0.73	0.04	3
BO	−1.23	−0.56	−0.93	4
PO	5.12	−0.29	3.54	2
TO	8.27	0.90	5.90	1
NO	−6.91	1.33	−4.66	6

Note: AO, SO, BO, PO, and TO represent ‘O’Neal’ grafted onto ‘Anna’, ‘Sharpblue’, ‘Baldwin’, ‘Plolific’, and ‘Tifblue’, respectively; NO represents own-rooted ‘O’Neal’.

**Table 4:** Effects of different rootstocks on the photosynthetic parameters in leaves of ‘O’Neal’ blueberry.

Grafted and Own-Rooted ‘O’Neal’	Net Photosynthetic Rate (*P_n_*)/μmol m^−2^ s^−1^	Transpiration Rate (*T_r_*)/mmol m^−2^ s^−1^	Stomatal Conductance (*G_s_*)/mmol m^−2^ s^−1^	Intercellular CO_2_ (*C_i_*)/μmol mol^−1^
AO	4.82 ± 1.51 c	0.38 ± 0.26 c	49.00 ± 14.42 b	87.23 ± 11.95 d
SO	8.42 ± 1.27 b	0.49 ± 0.04 bc	74.67 ± 9.27 b	109.00 ± 12.77 cd
BO	7.51 ± 1.30 b	0.50 ± 0.16 bc	94.00 ± 10.15 ab	141.67 ± 20.60 c
PO	11.13 ± 1.58 a	0.79 ± 0.27 b	140.00 ± 16.29 a	206.00 ± 11.36 b
TO	11.43 ± 1.80 a	0.88 ± 0.10 a	102.33 ± 14.57 ab	256.67 ± 28.36 a
NO	6.12 ± 1.43 bc	0.28 ± 0.06 c	46.33 ± 8.39 b	124.00 ± 24.00 c

Note: AO, SO, BO, PO, and TO represent ‘O’Neal’ grafted onto ‘Anna’, ‘Sharpblue’, ‘Baldwin’, ‘Plolific’, and ‘Tifblue’, respectively; NO represents own-rooted ‘O’Neal’. The data are means of three biological replicates ± standard deviation; Values followed by different lowercase letters within a column are significantly different at *p* < 0.05.

**Table 5:** Principal component scores and comprehensive evaluation of the anatomical structure in leaves and stems from grafted and own-rooted ‘O’Neal’.

Grafted and Own-Rooted ‘O’Neal’	Principal Component Score				Synthesis Score	Ranking
	P1	P2	P3	P4		
NO	−0.40	−0.34	−0.02	0.04	−1.13	3
AO	−2.98	0.99	0.83	0.04	−0.70	2
TO	3.38	2.42	−0.80	0.04	1.84	1

Note: AO and TO represent ‘O’Neal’ grafted onto ‘Anna’ and ‘Tifblue’ rootstocks, respectively; NO represents own-rooted ‘O’Neal’.

## Data Availability

Data will be made available on request.

## Supplementary Materials

The following supporting information can be downloaded at: https://www.mdpi.com/article/10.3390/plants13050625/s1, Table S1. Eigenvector, eigenvalue, variance contribution rate, and cumulative contribution rate of two principal components. Table S2. Correlation between photosynthetic parameters in leaves of grafted and own-rooted ‘O’Neal’. Table S3. Effects of different rootstocks on the photosythetic pigments in leaves of ‘O’Neal’ blueberry. Table S4. Eigenvector, eigenvalue, variance contribution rate, and cumulative contribution rate of four principal components. Table S5. Comprehensive evaluation on fruit quality traits of grafted and own-rooted ‘O’Neal’ (2021). Table S6. Principal component scores and comprehensive evaluation of fruit from grafted and own-rooted ‘O’Neal’ (2021). Table S7. Effects of different rootstocks on the plant height and crown width of ‘O’Neal’ blueberry. Table S8. Effects of different rootstocks on the photosynthetic parameters in leaves of ‘O’Neal’ blueberry (2021). Table S9. Correlation between photosynthetic parameters in leaves of grafted and own-rooted ‘O’Neal’ (2021). Table S10. Effects of different rootstocks on the photosythetic pigments in leaves of ‘O’Neal’ blueberry (2021). Table S11. Correlation between mineral elements in fruits and leaves of own-rooted ‘O’Neal’ (2021). Table S12. Correlation between mineral elements in fruits and leaves of ‘O’Neal’ grafted onto ‘Anna’ (2021). Table S13. Correlation between mineral elements in fruits and leaves of ‘O’Neal’ grafted onto ‘Plolific’ (2021). Table S14. Correlation between mineral elements in fruits and leaves of ‘O’Neal’ grafted onto ‘Tifblue’ (2021). Figure S1. The photographs of blueberry plants in 2022. Figure S2. Changes in phosphorus (P), potassium (K), calcium (Ca), and magnesium (Mg) concentrations in leaves of grafted and own-rooted ‘O’Neal’ (2021). Figure S3. Changes in iron (Fe), manganese (Mn), copper (Cu), zinc (Zn), and boron (B) concentrations in leaves of grafted and own-rooted ‘O’Neal’ (2021).
