# Supplementary material for: A Better Fruit Quality of Grafted Blueberry Than Own-Rooted Blueberry Is Linked to Its Anatomy
Source: Plants (Basel). 2024 Feb 24;13(5):625. doi: 10.3390/plants13050625 (PMC10935094; doi:10.3390/plants13050625)
Supplement: Supplementary file 1 [file plants-13-00625-s001.zip › plants-2878537-supplementary.pdf]

Table S1 Eigenvector, eigenvalue, variance contribution rate, and cumulative contribution rate of two principal components.

Quality index	Principal component 1	Principal component 2
Fresh weight per fruit	0.487	0.822*
Longitudinal diameter	0.828	-0.385
Transverse diameter	0.564*	-0.237
Total soluble solids	0.919*	-0.075
Titratable acidity	-0.883*	0.201
Solid : acid ratio	0.973*	-0.016
Anthocyanin	0.907*	0.329
Vitamin C	-0.980*	-0.017
Eigenvalue	5.595	1.035
Variance contribution rate (%)	69.932	12.938
Cumulative contribution rate (%)	69.932	82.870

Note: * indicates the absolute value of correlation coefficient > 0.5.

Table S2 Correlation between photosynthetic parameters in leaves of grafted and own-rooted 'O'Neal'.

Photosynthetic parameters	P_n	T_r	G_s	C_i
P_n	1			
T_r	0.740^{**}	1		
G_s	0.759^{**}	0.502^{*}	1	
C_i	0.797^{**}	0.740^{**}	0.598^{**}	1

Note: * and ** indicate significant correlation at the 5% and 1% levels, respectively.

Table S3 Effects of different rootstocks on the photosynthetic pigments in leaves of 'O'Neal' blueberry.

Grafted and own-rooted "O'Neal"	Chlorophyll a (mg/g)	Chlorophyll b (mg/g)	Chlorophyll a+b (mg/g)	Carotenoid (mg/g)	Chlorophyll a/b
AO	6.61±1.04a	1.11±0.69ab	7.12±0.48a	1.10±0.86a	4.46±2.79b
SO	5.66±0.70ab	1.88±1.27a	7.54±0.58a	1.21±0.30a	5.97±0.71ab
BO	7.16±1.14a	1.52±0.60ab	8.69±1.69a	1.46±1.07a	8.06±2.72a
PO	7.37±0.23a	1.16±0.07ab	7.53±0.15a	1.75±0.45a	6.08±0.34ab
TO	6.06±0.55a	1.40±0.66ab	7.47±1.15a	1.61±0.78a	5.32±1.08ab
NO	3.80±0.23b	0.93±0.29a	4.23±0.46b	1.89±0.31a	5.20±1.43ab

Note: AO, SO, BO, PO, and TO represent 'O'Neal' grafted onto 'Anna', 'Sharpblue', 'Baldwin', 'Plolific', and 'Tifblue' rootstocks, respectively; NO represents own-rooted 'O'Neal'. The data are means of three biological replicates±standard deviation;Values followed by different lowercase letters within a column are significantly different at $P<0.05$.

Table S4 Eigenvector, eigenvalue, variance contribution rate, and cumulative contribution rate of four principal components.

Characteristic index	Principal component 1	Principal component 2	Principal component 3	Principal component 4
Leaf thickness	0.969*	0.060	0.115	0.084
Upper epidermal thickness	0.895*	0.119	-0.050	0.275
Lower epidermal thickness	-0.442	0.560*	-0.190	0.529*
Palisade tissue thickness	0.631*	0.116	0.736*	-0.110
Sponge tissue thickness	0.949*	0.151	-0.112	0.038
Palisade tissue thickness/Sponge tissue thickness	-0.617*	0.286	0.682*	-0.190
Vessel diameter	-0.087	0.894*	--0.104	0.214
Vessel density	0.407	0.751*	-0.189	-0.293
Vessel wall thickness	0.249	-0.635*	0.165	0.528*
Conduit wall reinforcement	-0.063	-0.862*	-0.281	-0.390
Sapwood hydraulic conductivity	-0.414	-0.495	0.192	0.471
Eigenvalue	4.025	3.203	1.261	1.196
Variance contribution rate(%)	36.589	29.114	11.466	10.876
Cumulative contribution rate(%)	36.589	65.703	77.169	88.045

Note: * indicates the absolute value of correlation coefficient > 0.5.

Table S5 Comprehensive evaluation of fruit quality traits of grafted and own-rooted ‘O’Neal’（2021）.

Grafted and own-rooted “O’Neal”	Comprehensive evaluation value	Quality level
AO	0.34	Medium
PO	0.64	Good
TO	0.8	Excellent
NO	0.26	Medium

Note: AO, PO, and TO represent ‘O’Neal’ grafted on ‘Anna’, ‘Plolific’, and ‘Tifblue’, respectively; NO represents own-rooted ‘O’Neal’.

Table S6 Principal component scores and comprehensive evaluation of fruit from grafted and own-rooted ‘O’Neal’（2021）.

Grafted and own-rooted “O’Neal”	Principal component score		Synthesis score	Ranking
	P1	P2		
AO	-6.37	-1.31	-4.73	4
PO	3.67	-0.36	2.54	2
TO	6.41	-0.1	4.52	1
NO	-3.71	1.77	-2.33	3

Note: AO, PO, and TO represent ‘O’Neal’ grafted on ‘Anna’, ‘Plolific’, and ‘Tifblue’, respectively; NO represents own-rooted ‘O’Neal’.

Table S7 Effects of different rootstocks on the plant height and crown width of ‘O’Neal’ blueberry.

Rootstock	Plant height (cm)	Crown width (cm)
Anna	116.33±16.44c	111.44±20.80b
Sharpblue	127.33±17.06c	115.11±27.68b
Baldwin	157.11±21.77b	127.44±24.67ab
Plolific	180.22±21.8a	120.00±17.56ab
Tifblue	193.56±9.86a	141.33±27.83a
Own-rooted	150.22±17.28b	128.56±13.31ab

Note: The data are means of three biological replicates±standard deviation; Values followed by different lowercase letters within a column are significantly different at P<0.05.

Table S8 Effects of different rootstocks on the photosynthetic parameters in leaves of ‘O’Neal’ blueberry（2021）.

Grafted and own-rooted ‘O’Neal’	Net photosynthetic rate (P_n)/μmol m^{-2} s^{-1}	Transpiration rate (T_r)/mmol m^{-2} s^{-1}	Stomatal conductance (G_s)/mol m^{-2} s^{-1}	Intercellular CO_2 (C_i)/μmol mol^{-1}
AO	6.11±0.60b	0.68±0.11a	0.04±0.002b	130.67±19.50b
PO	10.28±0.59a	0.74±0.04a	0.06±0.006a	164.33±5.69a
TO	9.85±0.45a	0.75±0.05a	0.04±0.004b	137.33±14.57b
NO	5.89±0.94b	0.39±0.06b	0.01±0.001c	87.60±6.376.37c

Note: AO, PO, and TO represent ‘O’Neal’ grafted on ‘Anna’, ‘Plolific’, and ‘Tifblue’, respectively; NO represents own-rooted ‘O’Neal’. The data are means of three biological replicates±standard deviation;Values followed by different lowercase letters within a column are significantly different at P<0.05.

Table S9 Correlation between photosynthetic parameters in leaves of grafted and own-rooted ‘O’Neal’（2021）.

Photosynthetic parameters	P_n	T_r	G_s	C_i
P_n	1			
T_r	0.728**	1		
G_s	0.689*	0.806**	1	
C_i	0.711**	0.765**	0.895**	1

Note: * and ** indicate significant correlation at the 5% and 1% levels, respectively.

Table S10 Effects of different rootstocks on the photosynthetic pigments in leaves of ‘O'Neal’ blueberry（2021）.

Grafted and own-rooted ‘O’Neal’	Chlorophyll a (mg/g)	Chlorophyll b (mg/g)	Carotenoid (mg/g)	Chlorophyll a+b (mg/g)	Chlorophyll a/b
AO	6.26±0.45b	0.84±0.58b	1.91±0.88b	7.11±0.57b	9.64±0.94a
PO	8.54±0.19a	1.16±0.45a	2.30±0.37ab	9.70±0.57a	5.25±0.80a
TO	8.00±0.68a	1.06±0.13ab	2.35±0.57a	9.06±0.56a	6.93±1.83a
NO	5.90±0.46b	0.87±0.61b	1.99±0.80b	6.77±0.61b	7.83±3.96a

Note: AO, PO, and TO represent ‘O’Neal’ grafted on ‘Anna’, ‘Plolific’, and ‘Tifblue’, respectively; NO represents own-rooted ‘O’Neal’. The data are means of three biological replicates±standard deviation;Values followed by different lowercase letters within a column are significantly different at P<0.05.

Table S11 Correlation between mineral elements in fruits and leaves of own-rooted ‘O'Neal’（2021）.

Correlation coefficient		Fruit								
		P	K	Ca	Mg	Fe	Mn	Cu	Zn	B
Leaf	P	0.845**	0.839**	0.662*	0.668*	-0.23	0.946**	0.466	0.721**	0.232
	K	0.836**	0.918**	0.809**	0.817**	-0.004	0.936**	0.637*	0.875**	0.473
	Ca	0.326	0.359	0.413	0.249	-0.022	0.45	0.401	0.359	0.103
	Mg	0.616*	0.810**	0.922**	0.830**	0.324	0.750**	0.721**	0.878**	0.661*
	Fe	0.870**	0.966**	0.807**	0.868**	-0.043	0.949**	0.575	0.899**	0.517
	Mn	0.841**	0.801**	0.257	0.547	-0.492	0.838**	0.313	0.548	0.213
	Cu	0.726**	0.892**	0.583*	0.807**	-0.011	0.783**	0.581*	0.804**	0.652*
	Zn	0.564	0.825**	0.753**	0.876**	0.299	0.654*	0.626*	0.889**	0.876**
	B	0.554	0.699*	0.936**	0.756**	0.327	0.679*	0.556	0.827**	0.524

Note: * and ** indicate significant correlation at the 5% and 1% levels, respectively.

Table S12 Correlation between mineral elements in fruits and leaves of ‘O’Neal’ grafted onto ‘Anna’（2021）.

Correlation coefficient		Fruit								
		P	K	Ca	Mg	Fe	Mn	Cu	Zn	B
Leaf	P	0.702*	0.846**	0.685*	0.859**	0.24	0.772**	0.787**	0.867**	0.766**
	K	0.43	0.719**	0.837**	0.680*	0.516	0.523	0.587*	0.696*	0.920**
	Ca	0.186	-0.169	-0.554	0.168	-0.139	0.249	0.188	0.347	-0.117
	Mg	-0.041	0.223	0.597*	0.26	0.834**	0.072	0.138	0.335	0.898**
	Fe	0.065	0.307	0.552	0.376	0.794**	0.182	0.29	0.476	0.927**
	Mn	0.185	0.315	0.409	0.439	0.677*	0.303	0.373	0.577*	0.850**
	Cu	0.630*	0.772**	0.649*	0.795**	0.322	0.722**	0.735**	0.854**	0.809**
	Zn	0.744**	0.880**	0.701*	0.883**	0.2	0.806**	0.820**	0.880**	0.747**
	B	0.006	0.292	0.636*	0.272	0.812**	0.106	0.186	0.337	0.897**

Note: * and ** indicate significant correlation at the 5% and 1% levels, respectively.

Table S13 Correlation between mineral elements in fruits and leaves of ‘O’Neal’grafted onto ‘Plolific’（2021）.

Correlation coefficient		Fruit								
		P	K	Ca	Mg	Fe	Mn	Cu	Zn	B
Leaf	P	0.701*	0.857**	-0.025	0.691*	-0.755**	0.886**	0.246	0.853**	0.762**
	K	0.631*	0.806**	-0.06	0.614*	-0.806**	0.852**	0.131	0.799**	0.771**
	Ca	-0.54	-0.574	-0.861**	-0.729**	0.272	-0.376	-0.644*	-0.592*	-0.339
	Mg	-0.345	-0.115	-0.487	-0.247	-0.701*	0.032	-0.671*	-0.089	0.575
	Fe	-0.024	0.258	-0.288	0.125	-0.906**	0.333	-0.275	0.264	0.837**
	Mn	-0.191	0.067	-0.422	-0.107	-0.834**	0.19	-0.507	0.068	0.698*
	Cu	0.628*	0.820**	-0.071	0.625*	-0.818**	0.854**	0.19	0.800**	0.817**
	Zn	0.655*	0.831**	-0.07	0.659*	-0.791**	0.871**	0.197	0.831**	0.791**
	B	-0.306	-0.044	-0.205	-0.059	-0.801**	0.03	-0.446	0.023	0.736**

Note: * and ** indicate significant correlation at the 5% and 1% levels, respectively.

Table S14 Correlation between mineral elements in fruits and leaves of ‘O’Neal’ grafted onto ‘Tifblue’（2021）.

Correlation coefficient		Fruit								
		P	K	Ca	Mg	Fe	Mn	Cu	Zn	B
Leaf	P	0.893**	0.803**	0.041	0.646*	-0.064	0.706*	-0.1	0.367	0.045
	K	0.938**	0.877**	0.196	0.677*	-0.085	0.773**	-0.01	0.475	0.016
	Ca	-0.143	-0.341	-0.889**	-0.079	-0.152	-0.417	-0.253	-0.285	-0.219
	Mg	0.634*	0.464	-0.405	0.632*	0.227	0.395	-0.057	0.329	0.242
	Fe	0.775**	0.624*	-0.308	0.566	-0.088	0.484	-0.14	0.222	-0.033
	Mn	0.743**	0.581*	-0.37	0.599*	0.03	0.448	-0.049	0.261	0.067
	Cu	0.778**	0.681*	-0.154	0.861**	0.502	0.645*	0.241	0.622*	0.541
	Zn	0.740**	0.642*	-0.162	0.864**	0.580*	0.623*	0.293	0.661*	0.604*
	B	0.428	0.31	-0.445	0.399	0.133	0.232	0.118	0.066	0.065

Note: * and ** indicate significant correlation at the 5% and 1% levels, respectively.

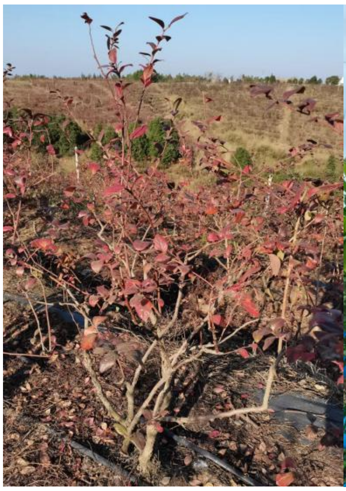
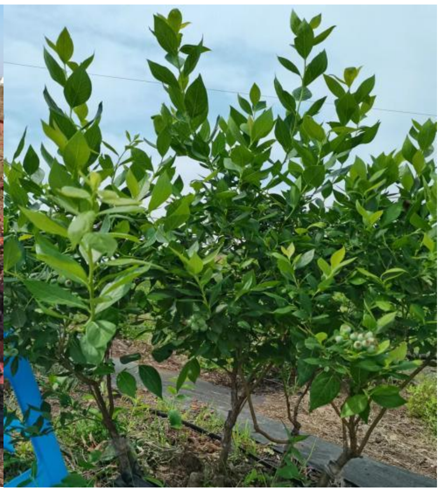

(AO1) (AO2)

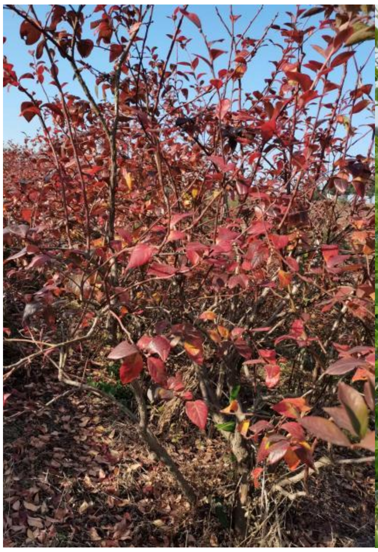
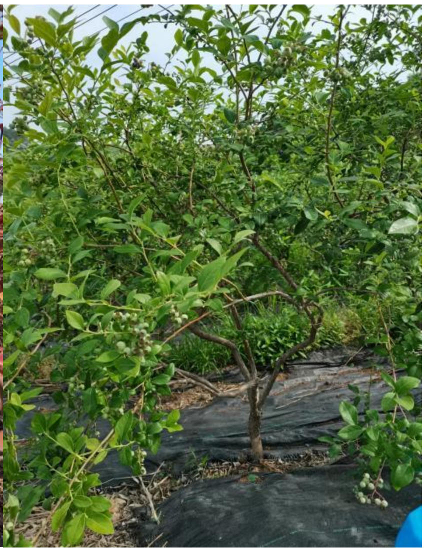

(TO1) (TO2)

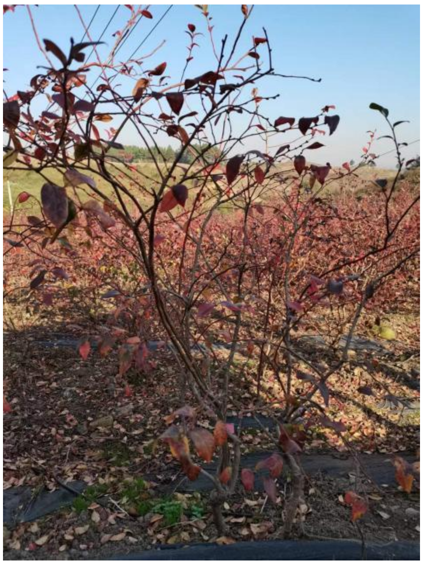

(NO)

Figure S1. The photographs of blueberry plants in 2022 Note: AO and TO represent ‘O’Neal’ grafted onto

‘Anna’ and ‘Tifblue’ rootstocks, respectively; NO represents own-rooted ‘O’Neal’.

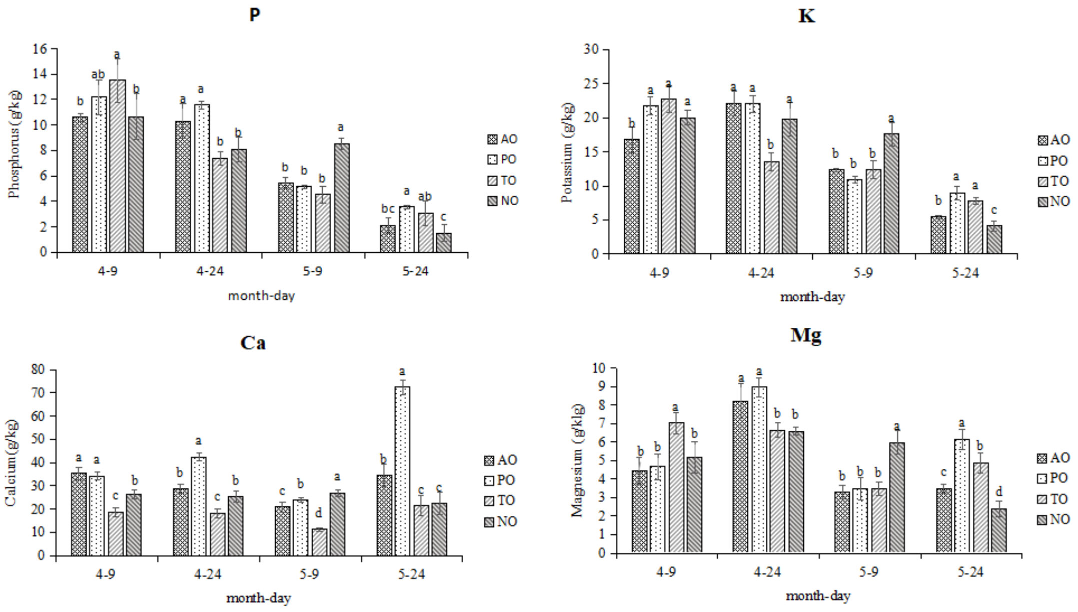

Figure S2. Changes in phosphorus (P), potassium (K), calcium (Ca), and magnesium (Mg) concentrations in leaves of grafted and own-rooted ‘O’Neal’ (2021). Note: AO, PO, and TO represent ‘O’Neal’ grafted on ‘Anna’, ‘Plolific’, and ‘Tifblue’, respectively; NO represents own-rooted ‘O’Neal’. Error bars were calculated based on three replicates; Different lowercase letters indicate significant differences with $P < 0.05$, as determined by Tukey’s test.

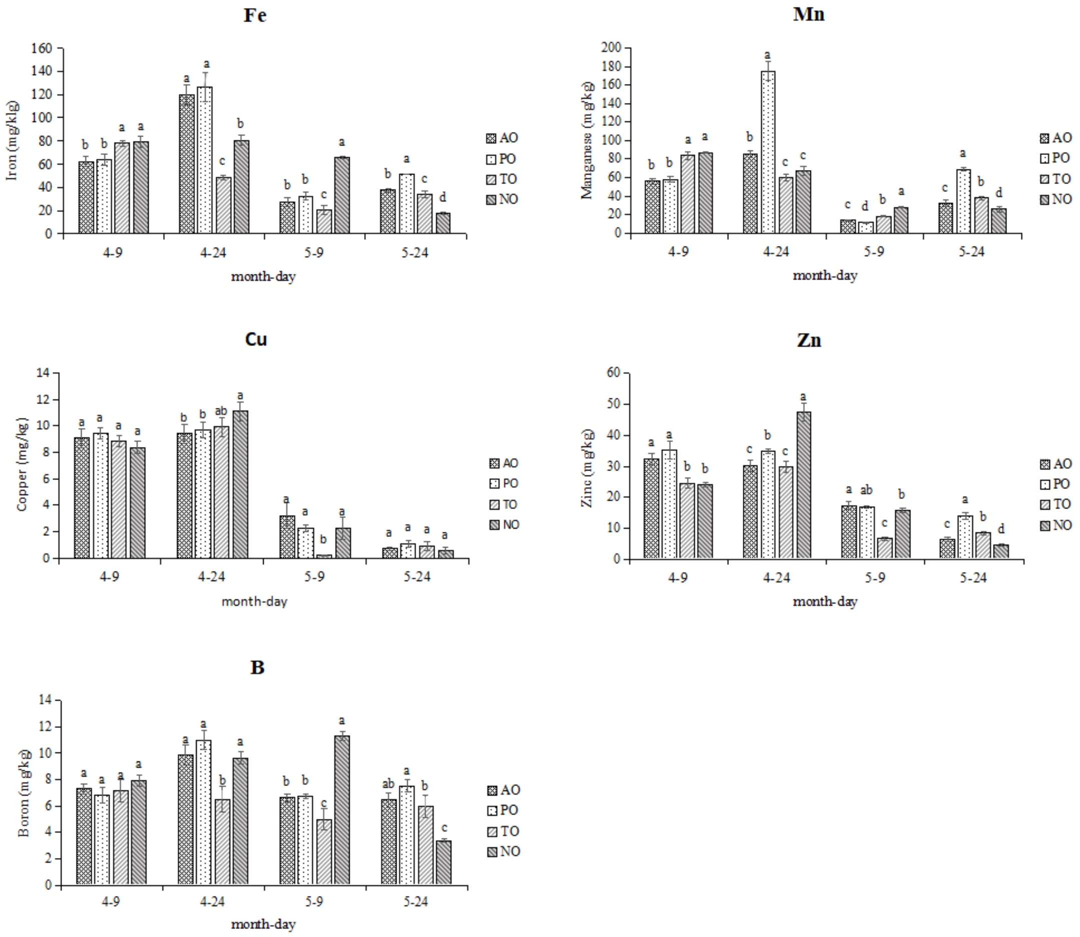

Figure S3. Changes in iron (Fe), manganese (Mn), copper (Cu), zinc (Zn), and boron (B) concentrations in leaves of grafted and own-rooted .O'Neal’ (2021). Note: AO, PO, and TO represent ‘O’Neal’ grafted on ‘Anna’, ‘Plolific’, and ‘Tifblue’, respectively; NO represents own-rooted ‘O’Neal’. Error bars were calculated based on three replicates; Different lowercase letters indicate significant differences with $P < 0.05$, as determined by Tukey’s test.
